# Case Report: Acute generalized exanthematous pustulosis with psoriasis successfully treated with Secukinumab

**DOI:** 10.3389/fimmu.2025.1648655

**Published:** 2025-11-19

**Authors:** Ji-Na Zheng, Xinying Liu, Yuling Shi, Jun Gu, Yu Gong

**Affiliations:** 1 Department of Dermatology, Shanghai Tenth People’s Hospital, Tongji University School of Medicine, Shanghai, China; 2 Institute of Psoriasis, Tongji University School of Medicine, Shanghai, China; 3 Rheumatology and Immunology Department, Shanghai Tenth People’s Hospital, Tongji University School of Medicine, Shanghai, China; 4 Department of Dermatology, Shanghai Skin Disease Hospital, Tongji University School of Medicine, Shanghai, China

**Keywords:** acute generalized exanthematous pustulosis, pustular psoriasis, secukinumab, IL36RN, case report

## Abstract

**Background:**

Acute generalized exanthematous pustulosis (AGEP) is a rare but severe cutaneous adverse reaction characterized by the abrupt onset of non-follicular sterile pustules on an erythematous and edematous background. These pustules typically appear within 24 to 48 hours after exposure to the causative drug, often localized to the face, trunk, and intertriginous area. Herein we firstly reported a 56-year-old man with severe plaque psoriasis who developed metamizole-induced AGEP during a clinical trial for BEBT-305 (HSP90 inhibitor) that was successfully treated with Secukinumab.

**Case summary:**

A 56-year-old man with a 40-year history of severe plaque psoriasis developed fever and self-administered metamizole. Within a few days, a progressive eruption appeared and worsened until hospital admission with a baseline PASI score of 50.3 three weeks later. The rash was characterized by diffuse erythema, intense pruritus and severe desquamation. Additionally, he developed edema in the lower extremities, and dense pustules appeared on his neck, inner thighs, and abdomen. The patient had no history of systemic treatment for psoriasis and had only used topical agents with limited benefit prior to presentation. Previously, the patient was enrolled in the “Heat shock proteins 90 inhibitors BEBT-305 Clinical Trial (CTR20222218)”. The patient took BEBT-305–200 mg orally daily for one month with no adverse effect. The patient had no prior medical history and no known drug allergies. Further tests and examination showed a high white blood cell count and C-reactive protein, abnormal liver function. The IL36RN and CARD14 gene tests were negative. A diagnosis of AGEP was made, allowing for the administration of Secukinumab 300 mg by subcutaneous injection. Sustained improvements were seen 1 week after the first dose of subcutaneous injection with the PASI score decreased to 11.8. Of note, there was resolution of the pustules and most erythema. After discharge, the patient received monthly injections of secukinumab 300 mg for seven months. PASI 100 response was achieved at 7 months. All skin lesions have cleared, with no recurrence to date.

**Conclusion:**

This case is the first case in which metamizole-induced AGEP with a history of psoriasis has been successfully treated with Secukinumab. Biologics should be considered in patients with AGEP, especially those with a history of psoriasis. This can benefit both diseases.

## Introduction

Metamizole-induced acute generalized exanthematous pustulosis (AGEP) is a rare but severe cutaneous adverse reaction characterized by the abrupt onset of non-follicular sterile pustules on an erythematous and edematous background. These pustules typically appear within 24 to 48 hours after exposure to the causative drug, often localized to the face, trunk, and intertriginous area (1). The eruption is often accompanied by fever and leukocytosis, with a peripheral blood neutrophilia. The drugs most frequently implicated in AGEP include antibiotics (such as beta-lactams, macrolides, and quinolones), antifungals (particularly terbinafine), and calcium channel blockers. Other less common triggers include antimalarials, analgesics, and contrast media (2). AGEP typically manifests rapidly, often within 24 to 48 hours following exposure to the inciting drug. The rash typically occurs within hours to days after the use of the inducing drug. In a case series study involving 97 patients with AGEP, the median time from drug exposure to symptom onset was as follows: 1 day for antibiotics and 11 days for all other drugs (3). This case report illustrates a patient with AGEP and severe plaque psoriasis who was successfully treated with Secukinumab.

## Case presentation

**Figure 1:**
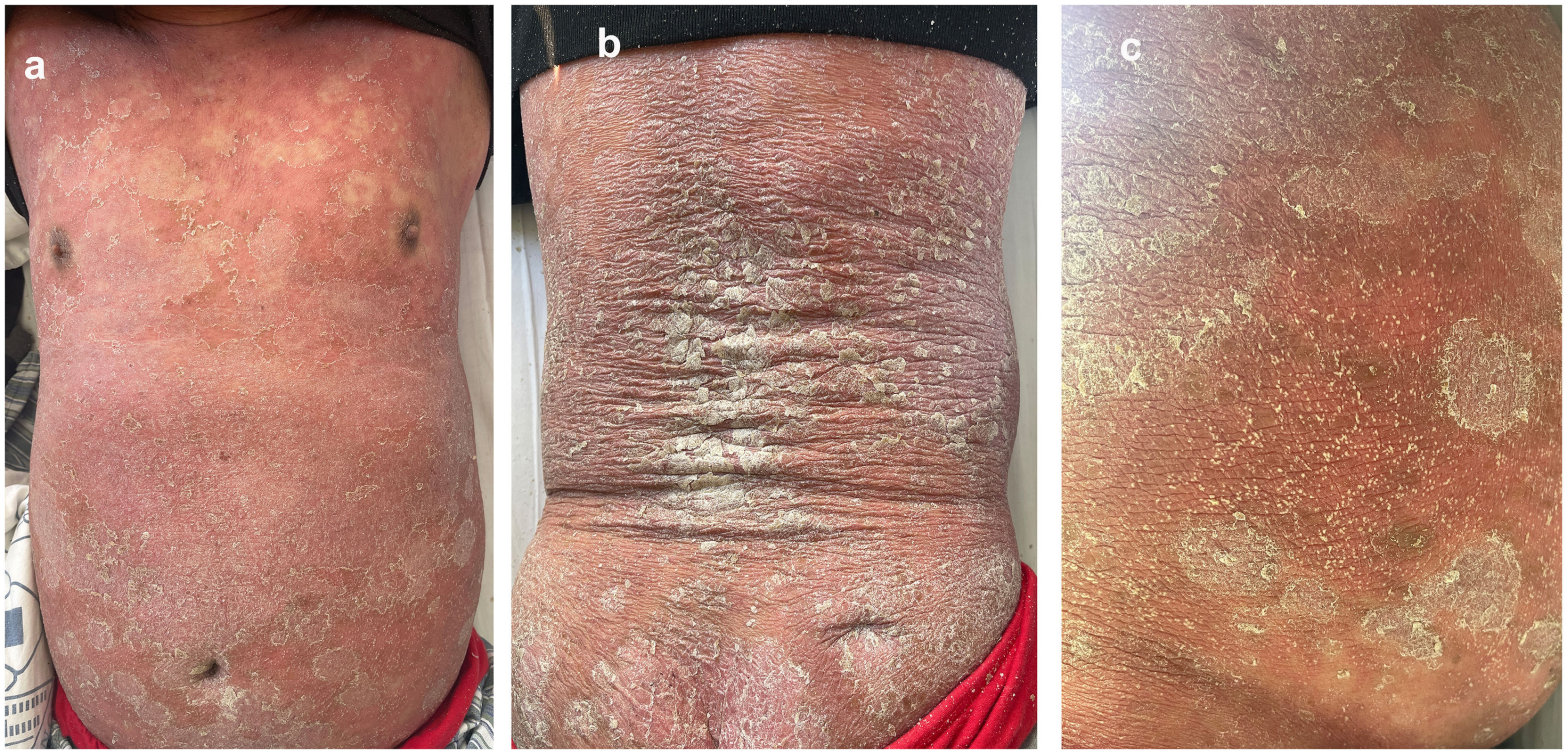
The patient present with diffuse erythema **(a)**, severe desquamation **(b)** and pustules appeared on his neck, inner thighs, and abdomen **(c)** after self-medicating with metamizole (PASI 50.3).

A 56-year-old man with a 40-year history of severe plaque psoriasis developed fever and self-administered metamizole. Within a few days, a progressive eruption appeared and worsened until hospital admission with a baseline PASI score of 50.3 three weeks later. The rash was characterized by diffuse erythema (**Figure 1a**), intense pruritus and severe desquamation(**Figure 1b**). Additionally, he developed edema in the lower extremities, and dense pustules appeared on his neck, inner thighs, and abdomen (**Figure 1c**). The patient also reported poor appetite, nausea, and dry retching. Previously, with the patient’s consent, the patient was enrolled in the “Heat shock proteins 90 inhibitors BEBT-305 Clinical Trial (CTR20222218)”. The patient took BEBT-305–200 mg orally daily for one month with no adverse effect. The patient had no prior medical history and no known drug allergies. He reported that it was first time taking metamizole. On admission, the patient presented with fever up to 39°C, leukocytosis (white blood cell count 14.48*10^9^/L), elevated C-reactive protein (CRP, 14.3 mg/L), and mildly elevated liver enzymes (ALT 53.6 U/L, AST 52.2 U/L). To exclude generalized pustular psoriasis (GPP), mutation analysis of the IL36RN and CARD14 genes was performed using Sanger sequencing. These genes were selected because pathogenic variants in IL36RN and CARD14 have been reported to be strongly associated with GPP, which represented an important differential diagnosis in this patient with a long history of psoriasis (4). The result showed that the IL36RN and CARD14 genes were negative.

**Figure 2:**
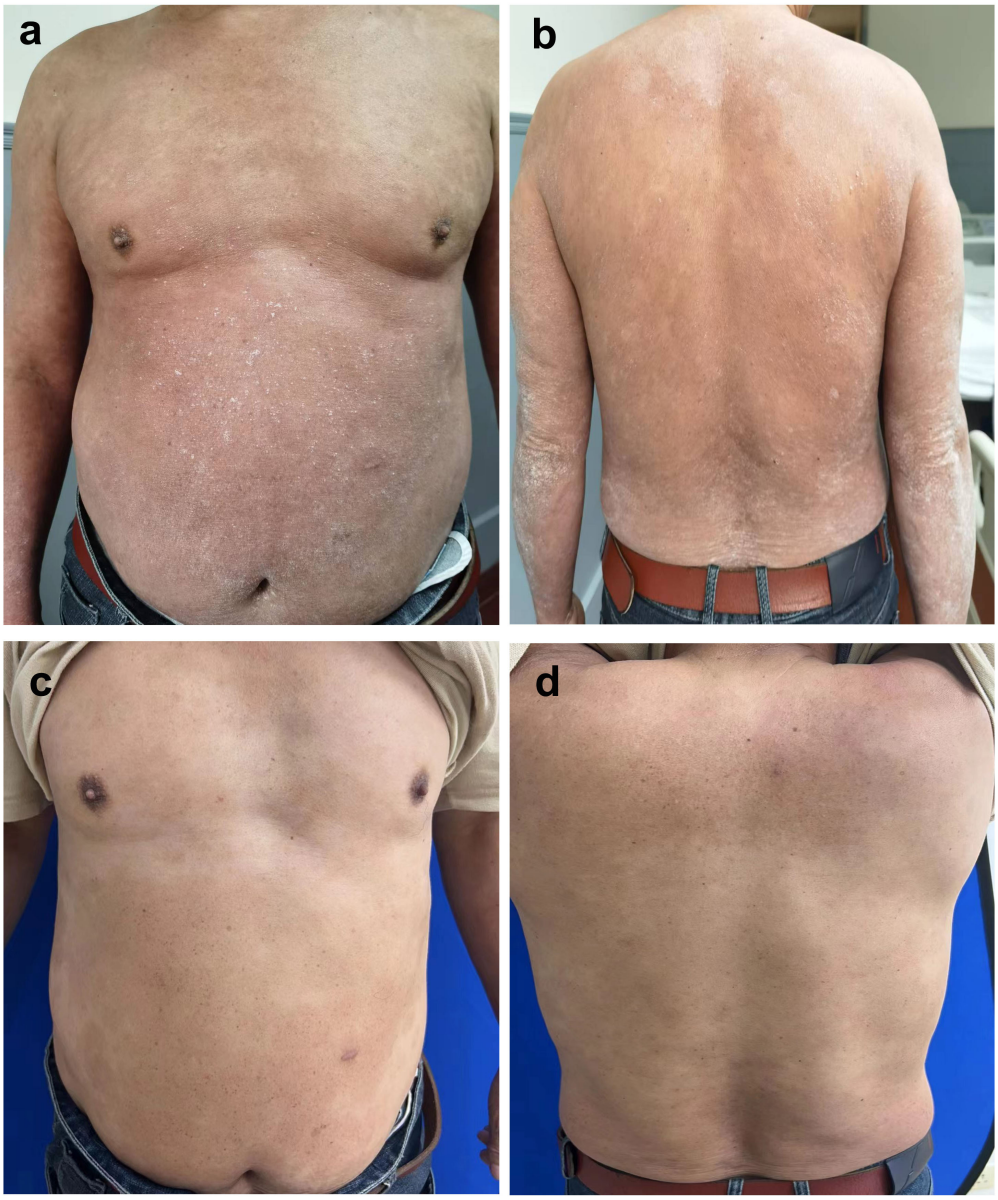
Sustained improvements were seen 1 week after the first dose of subcutaneous treatment (PASI 11.8). Most erythema disappeared **(a, b)**. Diffuse desquamation was reduced, resolution of pustules was observed after the subsequent doses of Secukinumab 300mg every one month for 7 months (achieved PASI 100) **(c, d)**.

Due to the patient’s long history of psoriasis, the key differential diagnosis was GPP. The worsening of skin lesions coincided with the self-medication of metamizole three weeks ago due to a fever, suggesting a potential drug-induced reaction. Negative results for IL36RN and CARD14 gene mutations, which are often associated with GPP, help differentiate AGEP from GPP (5). According to the EuroSCAR scoring system, which evaluates morphology, clinical course, and laboratory findings, the patient’s score was 8, indicating a definitive diagnosis of AGEP. The cornerstone of AGEP treatment is the prompt identification and discontinuation of the causative drug. Systemic corticosteroid treatment was not the first choice, because of the patient’s history of psoriasis. Recently, biologics have recently been reported to successfully treat AGEP (6–11). With the consent of the patient, we prescribed Secukinumab 300 mg by subcutaneous injection. Improvement was seen 1 day after the first dose of subcutaneous injection. Sustained improvements were seen 1 week after the first dose of subcutaneous injection, with the PASI score decreased to 11.8. Of note, there was resolution of the pustules and most erythema (**Figure 2a, b**). After discharge, the patient received monthly injections of Secukinumab 300 mg for seven months. The patient eventually achieved PASI 100 at 7 months of follow-up. All skin lesions have cleared (**Figure 2c, d**), with no recurrence to date. The patient remains under regular follow-up.

## Discussion

Diagnosing AGEP is a challenge owing to its rarity and overlap in symptoms with other conditions. Diagnosis of AGEP depends on clinical and histologic criteria. The AGEP validation score developed by the EuroSCAR group is a standardized tool used to classify and confirm cases of AGEP (1). This scoring system assesses patients based on three key criteria: morphology, clinical course and histology. Based on these criteria, patients can be classified as: definite, probable, possible and no AGEP. This scoring system helps in differentiating AGEP from other pustular diseases and ensures accurate diagnosis and appropriate management (**Supplementary Table S1**).

The differential diagnosis of AGEP is extensive, and we summarized several common diseases in **Supplementary Table S2**. AGEP is challenging to differentiate from GPP due to identical clinical and histopathologic findings, particularly those with a history of psoriasis. This case could help clinician distinguish AGEP from GPP and other pustular diseases.

Recent insights into the immunopathogenesis of AGEP have opened the door to novel therapeutic approaches. While the cornerstone of AGEP management remains the immediate discontinuation of the offending drug, emerging treatments have shown promise in severe or refractory cases. Targeted therapies, such as biologics including IL-17 antagonists (Secukinumab and Ixekizumab), IL-36 (Spesolimab) and TNF-alpha antagonists (Adalimumab and Infliximab), have recently been reported to successfully treat AGEP (6, 9–11). This case is the first report of metamizole-induced AGEP in a patient with psoriasis that was successfully treated with secukinumab, resulting in rapid resolution and no adverse effects. This case also indicates that IL-17A may play a pivotal role in the pathogenesis of both AGEP and psoriasis. Although the initiating mechanisms differ, both conditions share IL-17A–driven inflammatory pathways that promote neutrophil recruitment and pustule formation. Previous studies have demonstrated elevated IL-17A expression in the lesional skin and serum of AGEP patients, suggesting that IL-17A may represent a common downstream mediator between AGEP and psoriasis (6, 11). Therefore, IL-17A blockade with Secukinumab may provide therapeutic benefit for both diseases. Unfortunately, IL-17A immunohistochemical staining could not be performed in our case, as a skin biopsy was not obtained. Biologics should be considered in patients with AGEP, especially those with a history of psoriasis, as such treatment may benefit both diseases.

The strengths of this case include being the first report of metamizole-induced AGEP in a patient with psoriasis successfully treated with Secukinumab. This highlights the potential utility of secukinumab in complex clinical scenarios where conventional management may be insufficient. However, several limitations should be acknowledged. Although the clinical course and temporal relationship strongly suggest metamizole as the culprit drug, the absence of serum metabolite testing represents a limitation. Furthermore, this is a single case report, and the findings cannot be generalized to a broader patient population. Future studies with larger cohorts and longer follow-up will be needed to validate these observations.

## Conclusion

This case is the first case in which metamizole-induced AGEP with a history of psoriasis has been successfully treated with Secukinumab. However, further trials are needed to confirm the efficacy, safety, and long-term control of Secukinumab in AGEP patients.

## Data Availability

The raw data supporting the conclusions of this article will be made available by the authors, without undue reservation.
