# Supplementary material for: Case Report: Acute generalized exanthematous pustulosis with psoriasis successfully treated with Secukinumab
Source: Front Immunol. 2025 Nov 19;16:1648655. doi: 10.3389/fimmu.2025.1648655 (PMC12672486; doi:10.3389/fimmu.2025.1648655)
Supplement: Supplementary file 1 [file Table1.docx]

**Supplementary Table 1. Diagnostic score for acute generalized exanthematous pustulosis from EuroSCAR study**

| **Variable** | **Score** |
| --- | --- |
| **Morphology** |  |
| Pustules |  |
| Typical | +2 |
| Compatible with disease | +1 |
| Insufficient | 0 |
| Erythema |  |
| Typical | +2 |
| Compatible with disease | +1 |
| Insufficient | 0 |
| Distribution |  |
| Typical | +2 |
| Compatible with disease | +1 |
| Insufficient | 0 |
| **Course** |  |
| Mucous membrane involvement |  |
| Yes | -2 |
| No | 0 |
| Acute onset |  |
| Yes | 0 |
| No | -2 |
| Resolution within 15 d |  |
| Yes | 0 |
| No | -2 |
| **Fever** **≥38℃** |  |
| Yes | +1 |
| No | 0 |
| **Polymorphonuclear cells≥7000 cells/mm^3^** |  |
| Yes | +1 |
| No | 0 |
| **Histology** |  |
| Other disease | -10 |
| Not representative | 0 |
| Exocytosis of polymorphonuclear cells | +1 |
| Subcorneal and/or intraepidermal nonspongiform or NOS pustules with papillary edema or subcorneal and/or intraepidermal spongiform or NOS pustules without papillary edema | +2 |
| Spongiform subcorneal and/or intraepider  mal pustules with papillary edema | +3 |

**Score interpretation: ≤0 = no; 1-4 = possible; 5-7 = probable; 8-12 = definitive acute generalized exanthematous pustulosis. NOS, Not otherwise specified^1^**

1. Sidoroff A, Halevy S, Bavinck JN, et al. Acute generalized exanthematous pustulosis (AGEP)--a clinical reaction pattern. *Journal of cutaneous pathology* 2001;28(3):113-9. doi: 10.1034/j.1600-0560.2001.028003113.x [published Online First: 2001/02/13]
