# Supplementary material for: Case Report: Acute generalized exanthematous pustulosis with psoriasis successfully treated with Secukinumab
Source: Front Immunol. 2025 Nov 19;16:1648655. doi: 10.3389/fimmu.2025.1648655 (PMC12672486; doi:10.3389/fimmu.2025.1648655)
Supplement: Supplementary file 2 [file Table2.docx]

**Supplementary Table 2. The differential diagnosis of AGEP**

| Condition | Features | Differentiating Points |
| --- | --- | --- |
| Pustular Psoriasis | History of psoriasis, widespread sterile pustules overlaying scaly plaques, often with fever and other systemic symptoms.The antipsoriasis treatment is effective. | Histopathology shows subcorneal pustules with neutrophils, spongiform pustules of Kogoj, and Munro microabscesses. Onset can be acute or gradual, often triggered by withdrawal of systemic corticosteroids or infections^1-3^. AGEP is typically drug-induced, with a rapid onset after drug exposure, and resolves quickly upon discontinuation of the causative agent. |
| IgA pemphigus | Pruritic vesicles, pustules, and erythematous plaques, often with annular or serpiginous patterns, primarily involving the trunk and proximal extremities | Direct immunofluorescence (DIF) of perilesional skin: IgA deposits in the epidermal intercellular spaces^4^. AGEP is not associated with IgA deposition. |
| Dermatitis Herpetiformis | Intensely pruritic, vesicles, and pustules typically on extensor surfaces, associated with gluten sensitivity, celiac disease and gastrointestinal symptoms. | DIF showing granular IgA deposits at the dermoepidermal junction or the papillary dermis^5^. AGEP is not associated with gluten sensitivity. |
| Widespread pustular bacterial Infection | Systemic infection symptoms, erythema, widespread pustules containing bacteria. | Bacterial infections present with pustules that contain bacteria. AGEP pustules are sterile and there is no bacterial growth on culture^6^. |
| Eosinophilic Pustular Folliculitis (Ofuji Disease) | Recurrent crops of sterile pustules centered on hair follicles, pruritic, typically affecting the face, scalp, and trunk. | Histopathology shows eosinophilic infiltrate around hair follicles. AGEP typically shows neutrophilic infiltrate in pustules^7^. |
| Subcorneal Pustular Dermatosis (Sneddon-Wilkinson Disease) | Flaccid and superficial pustules on an erythematous base, that tend to coalesce, forming annular or circinate patterns, and primarily affecting flexural areas and trunk, recurrent episodes | Histopathology shows subcorneal pustules with neutrophils^8^. AGEP also shows neutrophilic pustules but usually has a more acute onset and drug association. |

**Reference**

1. Parisi R, Shah H, Navarini AA, et al. Acute Generalized Exanthematous Pustulosis: Clinical Features, Differential Diagnosis, and Management. *American journal of clinical dermatology* 2023;24(4):557-75. doi: 10.1007/s40257-023-00779-3 [published Online First: 2023/05/09]

2. Isom J, Braswell DS, Siroy A, et al. Clinical and histopathologic features differentiating acute generalized exanthematous pustulosis and pustular psoriasis: A retrospective series. *Journal of the American Academy of Dermatology* 2020;83(1):265-67. doi: 10.1016/j.jaad.2020.03.015 [published Online First: 2020/03/17]

3. Yamanaka-Takaichi M, Watanabe M, Comfere NI, et al. Differentiating generalized pustular psoriasis from acute generalized exanthematous pustulosis. *Journal of the American Academy of Dermatology* 2024;90(6):1289-91. doi: 10.1016/j.jaad.2024.01.080 [published Online First: 2024/03/14]

4. Kridin K, Patel PM, Jones VA, et al. IgA pemphigus: A systematic review. *Journal of the American Academy of Dermatology* 2020;82(6):1386-92. doi: 10.1016/j.jaad.2019.11.059 [published Online First: 2019/12/10]

5. Rodrigues F, Bachmeyer C. Coeliac disease and dermatitis herpetiformis. *Lancet (London, England)* 2018;392(10151):916. doi: 10.1016/s0140-6736(18)31503-4 [published Online First: 2018/09/22]

6. Nawas ZY, Tong Y, Kollipara R, et al. Emerging infectious diseases with cutaneous manifestations: Viral and bacterial infections. *Journal of the American Academy of Dermatology* 2016;75(1):1-16. doi: 10.1016/j.jaad.2016.04.033 [published Online First: 2016/06/19]

7. Nervi SJ, Schwartz RA, Dmochowski M. Eosinophilic pustular folliculitis: a 40 year retrospect. *Journal of the American Academy of Dermatology* 2006;55(2):285-9. doi: 10.1016/j.jaad.2006.02.034 [published Online First: 2006/07/18]

8. Watts PJ, Khachemoune A. Subcorneal Pustular Dermatosis: A Review of 30 Years of Progress. *American journal of clinical dermatology* 2016;17(6):653-71. doi: 10.1007/s40257-016-0202-8 [published Online First: 2016/06/29]
